# TGF-β1 expression is associated with invasion and metastasis of intrahepatic cholangiocarcinoma

**DOI:** 10.1186/s40659-015-0016-9

**Published:** 2015-05-21

**Authors:** Yijie Chen, Lin Ma, Qian He, Shaobo Zhang, Chenghua Zhang, Wei Jia

**Affiliations:** General Surgery, The 180th Hospital of People’s Liberation Army, Quanzhou, Fujian China; Department of General Surgery, the Eighth People’s Hospital of Qingdao, Qingdao, Shandong China; Department of Emergency Medicine, Tianjin Medical University General Hospital, Tianjin, China

**Keywords:** Intrahepatic cholangiocarcinoma, Transforming growth factor beta 1 (TGF-β1), Prognosis

## Abstract

**Background:**

Transforming growth factor (TGF)-β is involved in many physiologic processes, it often promotes metastasis, and its high expression is correlated with poor prognosis. In the present study, we analyzed the correlation between transforming growth factor beta 1 (TGF-β1) expression and prognosis in intrahepatic cholangiocarcinoma.

**Results:**

We examined the expression of TGF-β1 in 78 intrahepatic cholangiocarcinomas by immunohistochemistry and correlated the expression with clinicopathological parameters. TGF-β1 was expressed in 37 of 78 (47.4 %) intrahepatic cholangiocarcinomas. The expression of TGF-β1 was significantly correlated with lymph node metastasis, distant metastasis, and tumour recurrence. Patients with TGF-β1-positive tumours had significantly shorter survival time. In a multivariant analysis, the expression of TGF-β1 and the tumour stage were independent prognostic factors.

**Conclusions:**

Our data suggest that expression of TGF-β1 is a novel prognostic marker for intrahepatic cholangiocarcinoma.

## Background

Intrahepatic cholangiocarcinoma (ICC) is the second most common, primary hepatobiliary cancer after hepatocellular carcinoma. Despite overall advances in the diagnosis and treatment of patients with ICC, the prognosis of this disease still remains poor [1, 2]. One of the most important factors influencing the dismal prognosis of ICC is based on the late diagnosis at an advanced stage of disease, when tumour cell invasion into the blood and lymphatic vessels has resulted in metastatic spread and complete curative resection can no longer be achieved [3]. However, the molecular mechanisms underlying and driving metastasis remain unclear.

Transforming growth factor (TGF)-β is involved in various physiologic processes, such as wound healing, tissue development, and remodelling. TGF-β has also been implicated in many pathological conditions, including cancer, and has been shown to regulate a number of critical processes, such as angiogenesis, immune suppression, and cell migration [4–8]. TGF-β also induces a cytostatic response in most normal cell types and promotes metastasis in malignant tumour cells. Furthermore, high expression of TGF-β is correlated with poor prognosis in many malignant tumours [9–15].

There are three known mammalian isoforms of TGF-β, TGF-β1, −β2, and -β3, and all share significant structural and functional similarities [16]. Most malignant tumour cells exhibit positive TGF-β1 expression, with the most prominent expression in the plasma [14, 17]. Clinically, TGF-β1 is often elevated in the plasma of patients with malignant tumours. Preclinically, several models have shown correlations between TGF-β1 expression and increased tumourigenicity and increased invasion in many malignant tumours [8–10]. In this study, we analysed the expression of TGF-β1 in 78 ICC cases by immunohistochemistry, and investigated the association between TGF-β1 expression with clinicopathological parameters and patient survival.

## Results

### Expression of TGF-β1 protein in ICC

**Fig. 1:**
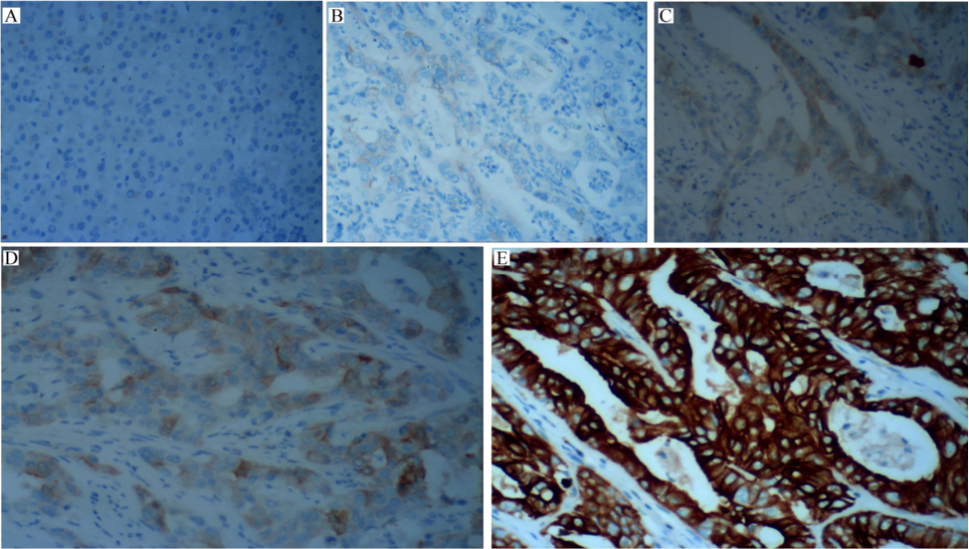
Immunohistochemical staining of TGF-β1 in ICC and normal liver. **a** TGF-β1 is not expressed in hepatocyte or bile duct. **b** ICC with negative TGF-β1 staining. **c** ICC with weak TGF-β1 staining. **d** ICC with moderate TGF-β1 cytoplasmic staining. **e** ICC with strong TGF-β1 cytoplasmic staining (×200)

In normal liver tissue, TGF-β1 was not expressed in hepatocytes or bile ducts (Fig. 1a). TGF-β1 was detected in 37 of 78 tumours (47.4 %). Among the 37 tumours positive for TGF-β1 expression, 14 tumours had weak staining and 23 tumours had moderate to strong staining. The tumours showed cytoplasmic staining of TGF-β1 (Fig. 1b–e). Generally, TGF-β1 expression showed considerable heterogeneity in the intratumoral distribution.

### Clinicopathologic significance of TGF-β1 expression in ICC

To evaluate whether TGF-β1 protein was associated with clinicopathological features of patients with ICC, we correlated immunohistochemical TGF-β1 staining results with major clinicopathologic features of ICC. As shown in Table 1, TGF-β1 expression was positively associated with lymph node metastasis, lymphovascular invasion, distant metastasis and tumour recurrence, but not with sex, patient age, tumour stage, and histologic differentiation or tumour size (pT).

**Table 1:** TGF-β1 expression and clinicopathologic parameters of ICC

	TGF-β1 expression		*p*-value
Gender	+	-	0.654
Male (n = 38)	18	20	
Female (n = 40)	19	21	
Age			0.453
<60 years(n = 31)	17	14	
≥60 years(n = 47)	20	27	
Stage			0.097
I–II (n = 37)	19	18	
III–IV (n = 41)	18	23	
Size			0.086
T1-T2 (n = 36)	16	20	
T3-T4 (n = 42)	21	21	
Lymph node metastasis			0.003
Yes (n = 20)	17	3	
No (n = 58)	20	38	
Lymphovascular invasion			0.045
Yes (n = 16)	10	6	
No (n = 62)	27	35	
Distant metastasis			0.012
Yes (n = 8)	8	0	
No (n = 70)	29	41	
Histologic dedifferentiation			0.145
Well differentiated (n = 42)	21	21	
Moderately differentiated (n = 15)	9	6	
Poorly differentiated (n = 21)	7	14	
Tumour recurrence			0.023
Yes(n = 47)	26	21	
No(n = 31)	11	20	

### Univariant survival analysis

**Fig. 2:**
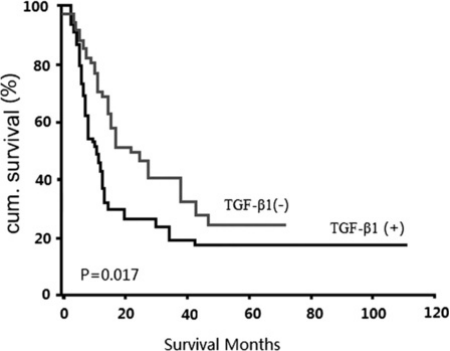
Univariant survival analysis of TGF-β1 in all 78 ICCs demonstrates that TGF-β1 positive tumours are associated with poor prognosis

In univariant survival analysis, we used the Kaplan–Meier method to calculate the cumulative survival curve and the differences in survival were accessed by the log-rank method. The conventional prognostic parameters including tumour size, nodal status, and disease stage reached significance for the overall survival. Patients with TGF-β1-positive tumours had a significant shorter survival than those with TGF-β1-negative tumours (P = 0.017; Fig. 2). The median survival of patients with TGF-β1-positive and − negative tumours was 7.3 and 16.6 months, respectively.

### Multivariant survival analysis

A multivariant progression analysis based on the Cox proportional hazard model was performed to analyse the independent value of each parameter predicting overall survival (Table 2). In this analysis, we included tumour size, nodal status, disease stage, and TGF-β1 expression. Tumour stage (*P* = 0.0000) and TGF-β1 expression (*P* = 0.001) proved to be independent prognostic factors for shortened overall survival in ICC.

**Table 2:** Multivariant survival analysis (Cox regression model) shows TGF-β1and tumor stage are independent prognostic factor

	Beta	Standard error	Wald	df	*p*-value
Stage	0.4973	0.1494	17.560	1	0.000
TGF-β1	0.6854	0.2981	5.853	1	0.001

## Discussion

In this study, we demonstrate that patients with ICC that express high levels of TGF-β1 have a higher chance of tumour recurrence, lymph node metastasis, lymphovascular invasion and distant metastasis than those with tumours that lack TGF-β1 expression. ICC is a highly aggressive tumour with generally poor prognosis characterized by intense desmoplastic stromal reaction and extensive vascular and perineural invasion.

In our series, immunohistochemical TGF-β1 expression is an independent prognostic indicator for ICC patients irrespective of vascular and lymphovascular characteristics. The high significance of our statistical data supports the hypothesis that TGF-β1 expression may interfere with the invasion process in ICC. TGF-β1 is an upstream effector of MMPs and VEGF, which could promote invasion, angiogenesis, and proliferation in many cancers [1–6], and thus could be a biological predictor of survival for ICC patients [6]. However, the current understanding of invasion and metastasis processes in ICC is poor and future studies should focus on identification of the specific molecular pathways involving TGF-β1 in these tumours.

The possibility of long-term survival of ICC patients depends on early diagnosis and the feasibility of a surgical resection in cases with localized disease [18, 19]. Patients not suitable for surgery generally face rapid disease progression with a survival rate of months according to the series [18]. Prognostic markers are needed to identify ICC patients with poor prognosis that might benefit from more aggressive surgical strategies [20].

Sulkowski et al. reported a high frequency of TGF-β1 positive colorectal cancers (up to 87 % of studied cases) with cytoplasmic accumulation both in malignant cells and inflammatory cells. Their results suggested that blockage of anticancer immunity is maintained by TGF-β1 and cell growth is not restricted by TGF-β1 as much in cancer cells as in normal intestinal epithelial cells [21]. According to the clinical-pathological reports on ICC, we also found that vascular invasion was a poor prognostic predictor of early tumour recurrence in ICCs (*P* < 0.05) and tumour invasion was a poor prognostic factor only in pancreatic cancers at univariate analysis [22]. Expression of TGF-β1 in our ICC series turned out to be a significant predictor of survival (*P* = 0.017) at univariate tests and an independent indicator of early tumour recurrence, regardless of vascular invasion.

## Conclusions

Our results indicate that TGF-β1 expression seems to represent a specific and independent neoplastic trait of ICC. Its immunohistochemical detection should provide a novel biological prognostic marker for ICC.

## Methods

### Ethical approval

The study was approved by The 180th Hospital of People’s Liberation Army, Quanzhou, and written informed consents were obtained from all participants prior to study entry.

### Patients

Between 2008 and 2012, 78 consecutive patients underwent surgical resection of ICC at General Surgery department at the 180th Hospital of the People’s Liberation Army. The group comprised 38 men and 40 women with a mean age of 64 years (range, 23.6–78.3 years). The distribution of TNM staging was as follows: stage I, *n* = 32; stage II, *n* = 8; stage III, *n* = 21, and stage IV, *n* = 16. At the end of follow-up, 49 patients were dead. A total of 64 patients were followed up at least 12 months or until death.

### Immunohistochemical analysis

Sections (4 μm) mounted on poly-L-lysine-coated slides were incubated for 30 min at 60 °C, deparaffinised by standard methods, and placed in 0.05 M Tris-HCI buffer, pH 7.2. Antigen retrieval was performed for 20 min in 10 mm sodium citrate buffer (pH 6) heated at 95 °C in a steamer, followed by cooling for 20 min. After blocking endogenous peroxidase activity with 0.3 % aqueous hydrogen peroxide for 5 min, the primary polyclonal rabbit anti-TGF-β1 antibody (DAKO, Carpinteria, CA, USA) was incubated with the sections at a final dilution of 2 mg/mL for 30 min. A control slide was incubated with Tris-HCI buffer substituted for the primary antibody. The DAKO LSAB™ kit/HRP was used for the detection of immunostaining. The sections were counterstained with Mayer’s haematoxylin. The staining intensity of TGF-β1 was semiquantitatively scored as negative, weak or moderate/strongly positive. A complete negative staining was scored as negative. A weak staining was defined as a minimal but unequivocal staining in less than 10 % of tumour cells. Stronger or more extensive staining was scored as moderate (10–50 %) or strong (≥50 %) positive expression. For statistical analysis, patients with weak and moderate/strong staining were lumped together as the TGF-β1-positive group in comparison with those with TGF-β1-negative tumours.

### Statistical analysis

Statistical analysis of TGF-β1 expression between specimen groups was carried out using the unpaired *Χ*^2^ test. Comparisons of clinicopathological factors (i.e. sex, age at diagnosis, histological type, pathological stage, differentiation, tumour stage, pathological stage, lymph node metastasis and metastasis) between positive—and negative-staining groups were performed using *Χ*^2^ tests. The log-rank test was used to compare survival distributions between the positive and negative staining groups, and Kaplan–Meier curves were plotted for the two groups. The clinical factors were accounted as being reasonable by fitting Cox’s proportional hazards models. Differences were considered statistically significant when the *P*-value was <0.05.

### Consent

Written informed consent was obtained from the patient for the publication of this report and any accompanying images.
